# Barriers and Facilitators to Implementing Post-Validation Surveillance of Lymphatic Filariasis in Pacific Island Countries and Territories: A Conceptual Framework Developed from Qualitative Data

**DOI:** 10.3390/tropicalmed11010027

**Published:** 2026-01-18

**Authors:** Harriet L. S. Lawford, Holly Jian, ‘Ofa Tukia, Joseph Takai, Clément Couteaux, ChoCho Thein, Ken Jetton, Teanibuaka Tabunga, Temea Bauro, Roger Nehemia, Charlie Ave, Grizelda Mokoia, Peter Fetaui, Fasihah Taleo, Cheryl-Ann Udui, Colleen L. Lau, Adam T. Craig

**Affiliations:** 1 UQ Centre for Clinical Research, Faculty of Health, Medicine, and Behavioural Sciences, The University of Queensland, Brisbane, QLD 4006, Australia; holly.jian@health.qld.gov.au (H.J.); colleen.lau@uq.edu.au (C.L.L.); adam.craig@uq.edu.au (A.T.C.); 2 Public Health Division, Ministry of Health, Nuku’alofa P.O. Box 59, Tonga; o.tukia@gmail.com (‘O.T.); uluakifalealeamasila@gmail.com (J.T.); 3 Wallis and Futuna Health Agency, Mata’utu 98600, Uvea, Wallis and Futuna; clement.couteaux@adswf.fr; 4 Ministry of Health and Human Services, Majuro 96960, Marshall Islands; cthein@rmihealth.org (C.T.); kjetton@rmihealth.org (K.J.); 5 Ministry of Health and Medical Services, Bairiki, Tarawa P.O. Box 67, Kiribati; ttabunga@mhms.gov.ki (T.T.); temea.bauro@mhms.gov.ki (T.B.); 6 Te Marae Ora—Ministry of Health, Avarua P.O. Box 109, Cook Islands; roger.nehemia@cookislands.gov.ck (R.N.); charlie.ave@cookislands.gov.ck (C.A.); 7 Niue Public Health Division, Halamahaga Rd, Alofi P.O. Box 40, Niue; grizelda.mokoia@gov.nu (G.M.); peter.fetaui@gov.nu (P.F.); 8 Ministry of Health, Port Vila P.O. Box 177, Vanuatu; taleof@who.int; 9 Bureau of Public Health, Palau Ministry of Health, Koror 96940, Palau; tmong.udui@palauhealth.org

**Keywords:** lymphatic filariasis, post-validation surveillance, Pacific Island Countries and Territories, neglected tropical diseases, barriers and facilitators, thematic analysis, operational guidance, health systems integration, capacity strengthening, disease elimination

## Abstract

Eight Pacific Island Countries and Territories (PICTs) have been validated by the World Health Organization (WHO) as having eliminated lymphatic filariasis (LF) as a public health problem. WHO recommends that these countries implement post-validation surveillance (PVS) to ensure resurgence has not occurred. Some PICTs proactively conducted LF PVS even in the absence of specific recommendations or best-practice guidelines at the time of implementation. We aimed to explore the barriers and facilitators to implementing LF PVS in PICTs, with a view to informing context-specific strategies and regional guidelines. Key informant interviews were held between March and September 2024 with 15 participants involved in LF and/or neglected tropical disease surveillance. Transcripts were analysed thematically using a generalised deductive approach. A conceptual framework was developed to summarise themes with two main streams of barriers identified. Stream One Barriers included limited awareness of, and guidelines for, PVS requirements and competing national health priorities. Stream Two Barriers included cost, resource, and logistical barriers to conducting PVS. Participants called for clearer, contextually tailored guidelines, improved communication from WHO, and integration within existing systems. This study highlights the urgent need for operational guidance, policy advocacy, and capacity strengthening to ensure sustainable LF PVS in PICTs. Incorporating local context and leveraging existing health structures will be essential to prevent disease resurgence and maintain gains achieved through elimination programmes.

## 1. Introduction

Lymphatic filariasis (LF) is a neglected tropical disease (NTD) transmitted by mosquitoes and caused by three species of filarial worm (*Wuchereria bancrofti* [90% of global cases]; *Brugia malayi*; or *Brugia timori*) [1]. *Wuchereria bancrofti* is the dominant species of parasite causing LF in the Pacific Island Countries and Territories (PICTs) [2] transmitted by *Aedes* mosquitoes [3].

The Pacific Programme to Eliminate LF (PacELF) was established in 1999 with the aim of eliminating LF as a public health problem in the 22 PICTs of the Western Pacific Region by 2020 [4]. In 2000, the Global Programme to Eliminate Lymphatic Filariasis (GPELF) was launched by the World Health Organization (WHO). GPELF’s aim was to (i) interrupt the transmission in LF-endemic districts through community-wide mass drug administration (MDA) of anthelminthic medicines, and (ii) manage the morbidity and prevent the disability of affected populations (MMDP) by 2020 [5].

The validation of elimination of LF as a public health problem is an official WHO process to confirm that countries have achieved the required elimination thresholds. The pathway to WHO validation of elimination of LF as a public health problem is well defined. Following baseline mapping, implementation units undertake annual MDA to interrupt transmission. After a minimum of five effective rounds of MDA, Transmission Assessment Survey (TAS) 1 is conducted to determine whether infection levels have fallen below WHO-defined thresholds, allowing for the cessation of MDA. Post-MDA surveillance (TAS 2 and TAS 3) conducted at 2–3 year intervals verifies whether the interruption of transmission has been sustained. To obtain the validation of elimination as a public health problem, countries must submit a dossier that includes (i) survey results demonstrating a sustained reduction of LF infection below target thresholds (<1% LF antigen [Ag]-prevalence in areas where *Aedes* are the main vector); (ii) plans for MMDP; and (iii) a commitment for post-validation surveillance (PVS) [6]. The main objectives of PVS are to detect and respond to any recrudescence or reintroduction of LF (and thereby prevent disease resurgence) and provide additional information to confirm the elimination of LF transmission [6].

Of note, the term ‘elimination as a public health problem’ refers to reducing the disease burden to below a WHO-defined threshold within a specific geographic area (requiring continued monitoring to ensure prevalence levels remain below the threshold). In contrast, ‘elimination of transmission’ refers to zero incidence in a defined geographic area (requiring ongoing measures to prevent reintroduction) and ‘eradication’ which denotes the permanent global reduction of the infection to zero, after which intervention measures are no longer needed [7].

**Figure 1:**
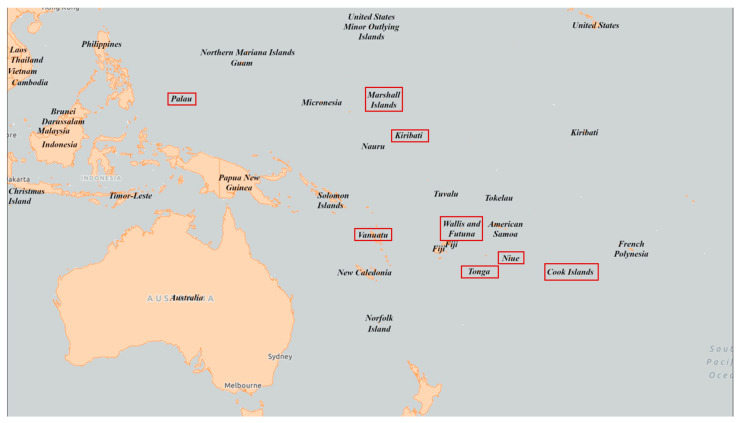
Map of Pacific Island Countries and Territories that have eliminated lymphatic filariasis as a public health problem, indicated by red boxes, as of June 2025. Basemap data © Esri/Natural Earth; annotations by authors.

Due to the concerted effort of PacELF, the GPELF, PICT Ministries of Health (MOH) and their partners, significant gains have been made towards LF elimination. To date, eight previously LF-endemic PICTs (the Cook Islands, Kiribati, Republic of the Marshall Islands, Niue, Palau, Tonga, Vanuatu, and Wallis and Futuna [Figure 1]) have eliminated LF as a public health problem [8]. New milestones and targets for LF elimination beyond 2020 have been established. The *WHO: Neglected Tropical Diseases Roadmap 2030* [9] suggests that by 2030 all endemic countries (i) complete MDA programmes; (ii) implement post-MDA surveillance or PVS (if they have reached elimination as a public health problem); and (iii) implement a minimum package of care to manage patients with lymphoedema or hydrocele (MMDP) [10].

Although LF PVS has been conducted in several countries [11,12,13,14,15], at the time of implementation there was limited official guidance available for how to conduct LF PVS, particularly the frequency and length of time PVS should be conducted, measurable thresholds to determine whether LF elimination has been sustained, or how ‘signals’ (e.g., positive rapid antigen tests, lymphedema) of persistent LF transmission should be dealt with [16]. We note that the *WHO 2nd Edition of the LF Monitoring & Evaluation manual* [7] was released in July 2025, which includes information regarding the implementation, timing, and duration of PVS, as well as the use of surveillance data.

On the 31 August 2023, the inaugural Regional Coalition for Operational Research on NTDs (COR-NTD) Meeting for the Pacific Islands (Regional COR-NTD Meeting) was held in Sydney, Australia [17]. The goal of the Meeting was to “bring together experts and stakeholders to share experiences and discuss key priorities for the PICT NTD community, with a view to moving the research agenda forward.” A total of 130 participants from 21 countries (including 11 PICTs) attended the meeting and two breakout sessions on LF were held. These breakout sessions aimed to instigate robust debates, inter-country lesson sharing and learning, and identify operational research questions related to LF that, once answered, would inform evidence-based policy and programme development. Breakout Session One focussed on LF surveillance in countries that have not yet reached the validation of elimination of LF, whilst Breakout Session Two discussed potential PVS strategies and their operational feasibility in PICT contexts.

Breakout Session Two was attended by NTD programme representatives from Tonga, Kiribati, the Cook Islands, French Polynesia, and Timor-Leste. During the discussion it became evident that some PICTs had taken the initiative and conducted PVS in the absence of formal global mandates or established best practice guidelines. The knowledge derived from the experiences of these proactive, early implementers of PVS is very valuable. If harnessed, the experiences could play a pivotal role in developing and contextualising regional LF PVS strategies. Similarly, understanding the barriers as to why some countries who have achieved validation of LF elimination as a public health problem have not conducted PVS will be helpful to determine where support and guidance are required to ensure LF surveillance continues in post-validation settings. Further, highlighting inhibiting factors outside of NTD programme officers’ direct control is crucial for LF PVS implementation.

This study aimed to identify the barriers and inhibitors to PVS implementation among PICTs who have achieved the validation of LF elimination as a public health problem. The objectives were to (i) develop a conceptual model to summarise barriers to PVS implementation in PICTs; (ii) explore variation in barriers across PICT settings; and (iii) identify key considerations and examples of best practice for future implementation of LF PVS in the PICT context.

## 2. Materials and Methods

***Ethical approval and consent process:*** Ethics approval was granted by The University of Queensland Human Research Ethics Committee (Project number: 2024/HE000011). Introductory emails were sent to participants, which explained the objectives of the study, its rationale, and any risks and benefits. A standard participant information sheet and consent form was attached, with the consent form requested to be returned before the commencement of interviews. Participants retained their information sheet, and a signed consent form was kept by both the participant and the study team. Verbal confirmation of consent was obtained at the start of in-depth interviews and participants were advised that they could revoke their consent at any time without any prejudice. Permission for the interviews to be audio-recorded was obtained from each participant.

We did not anticipate any risk of harm or psychological distress to the participants. The research team had prior professional collaborations within the region, which facilitated trust and open discussion. To minimise power imbalances, interviews were conducted in a non-evaluative manner, emphasising shared learning rather than programme performance. Participants were encouraged to reflect on system-level challenges rather than individual or country-specific shortcomings. Given the small size of participating jurisdictions, care was taken to minimise deductive disclosure risk. Quotes were deidentified, role-specific details were removed where necessary, and findings were presented in aggregate across settings to reduce the likelihood of identifying individuals or countries.

***Participant recruitment:*** Potential participants from the eight PICTs that had eliminated LF as a public health problem were identified through attendance at the Regional COR-NTD Meeting and through existing professional networks of the research team. Contact details were obtained through publicly available institutional directories, MOH channels, and existing professional correspondence. Identified individuals were emailed a link to an online screening questionnaire developed on the Qualtrics^®^ platform. These participants were those involved in LF surveillance activities leading up to validation and/or currently responsible for LF and/or NTD surveillance in their respective country or territory. These individuals were purposively selected to ensure representation across all eight PICTs and to capture a range of roles. A total of 15 respondents completed the screening questionnaire (summarised in Table 1) and were subsequently approached for in-depth interviews.

***Data collection:*** Qualitative in-depth interviews were conducted using an interview guide (see Appendix A) and undertaken online via Zoom or in-person where possible. Interviews were semi-structured with prompts as required and took approximately one hour. Participants were encouraged to speak openly regarding their experiences, but discussion was steered towards the project aims. All interviews were recorded and transcribed by an external consultant. Transcriptions were manually cross-checked and edited where needed. Sample adequacy was guided by an information power approach rather than numerical saturation. Participants were highly specialised informants with direct responsibility for LF or NTD surveillance within small health systems. Adequacy was assessed based on the richness of data, recurrence of themes across settings, and the study’s focused aims, rather than the total number of interviews conducted.

***Data analysis:*** Prior to coding, transcripts were checked for accuracy against the recordings and deidentified. Transcribed interviews were coded using a generalised deductive approach [15]. This method was chosen because it enabled us to assess how the new data aligned with and added nuance to the existing knowledge on enablers and barriers to LF PVS, while also supporting the development of setting insights to inform our contextualised conceptual framework. A deductive structure also facilitated comparison across sites by applying consistent thematic categories. Coding was managed using NVivo (Version 14, QSR International, Melbourne, Australia) and was initially conducted by the lead researcher (HL) using a structured deductive framework informed by the study objectives. Coding was iteratively reviewed with the senior co-author (AC), with discrepancies discussed and resolved through consensus. A codebook was developed and refined throughout analysis to ensure consistency across transcripts. A summary version of the codebook can be made available upon request. Emergent themes were grouped and presented to best respond to the project’s aims. Analysis was focused on country-level activities, rather than the individual.

***Patient and public involvement:*** No patients or members of the public were involved in the research design, analysis, nor dissemination of the findings. The study findings were shared with participants to ensure both accuracy and culturally appropriate framing.

## 3. Results

A total of 15 participants from eight PICTs were interviewed between 8 March 2024 and 29 September 2024. The mean length of interview was 60 min (range 40–80 min).

**Figure 2:**
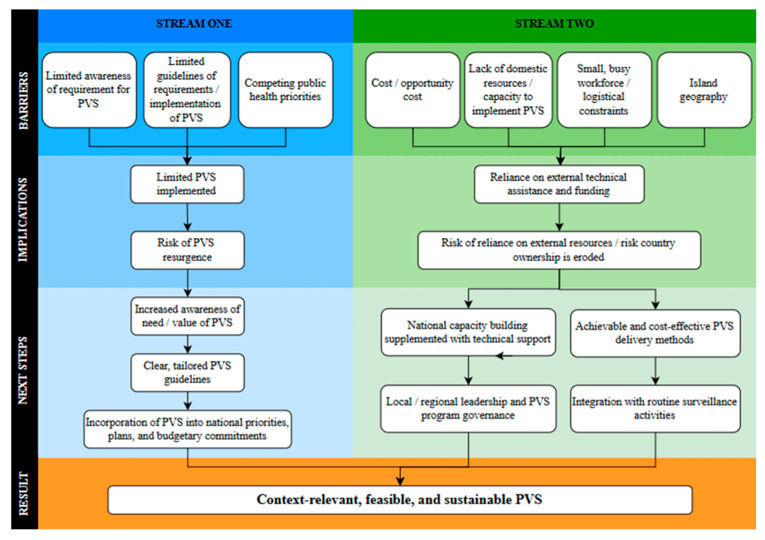
A conceptual framework of perceived barriers to post-validation surveillance of lymphatic filariasis in Pacific Island Countries and Territories.

As shown in Figure 2 (and supported by the qualitative analysis of in-depth interviews summarised below), we identified seven key themes related to barriers to LF PVS implementation in PICTs. These were the following: (1) limited awareness of the requirement for PVS; (2) limited guidelines on requirements/implementation of PVS; (3) competing public health priorities; (4) cost/opportunity cost; (5) limited domestic resources and workforce capacity; (6) small, overburdened workforce and logistical constraints; and (7) island topography and accessibility issues.

These barriers were classified into two streams: Stream One Barriers related to barriers caused by limited awareness and guidelines on PVS requirements as well as competing national health priorities, and Stream Two Barriers related to cost (financial barriers), resource (including staffing, time, and infrastructure), and logistical barriers. The barriers, implications of these barriers, and possible next steps for each stream are presented below.

Themes were grouped into two analytical streams based on the level at which barriers primarily operated. Stream One Barriers reflected the challenges related to awareness, policy, prioritisation, and guidance, while Stream Two Barriers reflected the operational constraints related to financing, workforce capacity, logistics, and geography.

***Stream One Barriers: Limited awareness of, and guidelines for, PVS requirements and competing national health priorities***

A key barrier captured by several PICTs was limited awareness of the requirements for LF PVS once elimination as a public health problem had been achieved. Participants reported that the processes and obligations for LF PVS were poorly communicated to national health staff and decision-makers, and, in several cases, health personnel only became aware of LF PVS requirements after attending international workshops or meetings, underscoring pivotal gaps in communication, information flow, and guidance from global partners.

*“The process and requirements for PVS after the declaration of LF elimination was not well communicated to us, especially to the decision makers to make sure it is a priority and to be established and conducted as recommended.”*

The importance of clear communication using non-technical terminology was highlighted; in many PICT languages, there is only one word for “elimination” and “eradication”, leading to the presumption that LF surveillance activities were no longer required because there was no more disease. This sentiment was captured by one participant who stated that no diagnostic testing for LF was conducted in their country “because LF had been eliminated” and therefore, ongoing surveillance was not considered necessary. The importance of distinguishing between disease “elimination” as a public health problem and “eradication” was further captured by one participant who said:

*“But elimination is defined by prevalence lower [than] one per cent. It’s not eradicated…If we don’t improve our communication, I think it’s difficult to transmit the message that LF is not eradicated. We have still transmission.”*

Confusion over the purpose and expectations of PVS was widespread due to the absence of clear, timely, and dedicated LF PVS guidelines. This left local authorities unsure about how to structure PVS, captured by one participant who stated that PVS efforts felt like “putting the cart before the horse” with guidance arriving too late to meaningfully inform an LF PVS strategy. This uncertainty extended to the technical aspects and decision-making; questions were raised around interpretation of diagnostic test results and how to differentiate between active and inactive infections, recommended treatment for those who test positive, and whether reliance on clinical presentation (passive surveillance) was sufficient or if active surveillance (e.g., surveys) should be in place, such as entomological surveillance (as suggested by one participant).

A recurring barrier to implementing LF PVS that was cited was the competition with other pressing public health concerns. Across PICTs, respondents consistently mentioned the burden of, and political focus on, non-communicable diseases (NCDs) as having shifted national attention and health priorities from communicable diseases. In the same vein, one participant explained that COVID-19 consumed all available resources for an extended period, and this has had a lasting impact on both the functionality and resources provided to other health programmes, including NTD programmes. A result of this shift away from investment in, and focus on, communicable diseases was limited awareness of, and training around, LF among front-line healthcare workers and the general population, contributing to the under-recognition and under-reporting of potential cases.

Across PICTs, these informational and systemic barriers have led to fragmented, delayed, or entirely absent implementation of LF PVS, preventing the detection of any persistent LF transmission, increasing the risk of resurgence and establishment of endemic transmission, and potentially reversing decades of work and financial investment. To address these challenges, participants highlighted the need for increased awareness of the purpose and value of LF PVS, underpinned by clear, dedicated guidelines. Tailored, context-specific guidelines aligned with local cultural practices were seen as essential to ensuring feasibility and sustainability of LF PVS in local contexts; such ideas cited by one participant included placing mosquito traps in areas where kava is consumed during peak mosquito activity times and developing region-specific diagnostic thresholds using different biomarkers, e.g., eosinophilia cut-offs. These observations underscore the need for guidelines that reflect context and community-specific patterns of exposure and disease.

Policy improvements, financial investment, and integration with existing health systems is also necessary. Given the perception among both communities and policymakers that LF is no longer a concern following elimination, participants expressed a strong need to re-engage decision-makers and communities and raise awareness about the need to prioritise PVS as a critical public health activity by reintroducing LF and other NTDs into national health agendas. This was captured by one participant who stated:

*“Because LF had been eliminated, they think it’s no longer a problem and it’s not in their agenda. So, I think this is the right time for us to get these things moved forward and make sure it’s communicated well to the decision makers to be part of the health priorities.”*

However, incorporating PVS into national plans and budgets remains a significant challenge; a clear gap emerged between the political priorities and financial commitments wherein, although public health is a priority, the majority of funding is allocated to salaries, leaving limited resources for operational activities. Participants also pointed to the outdated policy and legislative frameworks that failed to include LF and other NTDs in notifiable disease lists, underscoring the need for policy revision and evidence-based advocacy to elevate these issues in national planning and resource allocation. Another participant also emphasised structural limitations, calling for education and institutional reform to better support and sustain LF PVS.

***Stream Two Barriers: Cost, resource, and logistical barriers to conducting PVS***

Participants consistently highlighted challenges regarding limited domestic resources and small, over-burdened national workforce capacity as critical barriers to implementing LF PVS. Participants reported that national programmes struggled to secure resources to establish and sustain disease surveillance activities, let alone for a disease that is perceived to be eliminated. Notably, such challenges can lead to disempowerment and dependence on external development partners.

*“The balance of resources is very important, and we are small, small territories. And we have not a lot of resources, and it’s very, very complicated to improve surveillance of all communicable diseases. And so, we, when we have a problem, we treat the problem. But after, yes, it’s very complicated.”*

Despite these barriers, one PICT had proactively integrated PVS into an existing, broader NTD serosurvey; however, limitations in funding had prevented the expansion of surveillance into additional provinces that were known LF hotspots in previous surveys.

A recurring issue was the lack of access to LF rapid antigen tests, both in terms of availability and timely procurement. Four PICTs reported either no access to LF rapid antigen tests or significant delays in obtaining them. In one PICT, microfilaria laboratory tests were the only diagnostic test available, noting that these came with additional operational caveats including finding trained laboratory personnel to prepare and read slides. Two PICTs elaborated on the logistical and financial strain of acquiring and distributing LF rapid antigen tests, including the challenges of shipment delays, short shelf lives, and inappropriate packaging for low-prevalence settings. One PICT specifically described how the standard packaging of LF rapid antigen tests led to wastage in settings that required only a handful of tests. Some participants noted that even when tests are received, their expiration date often left only a few months for use, undermining the effort to build routine and sustainable surveillance.

*“Because, for us in the Pacific, one-year, two-year lifespan is too short. Because to receive [test kits] is one thing and then to ship it to the province and province to the health facilities … And by the time you reach there, it’s only left two, three months [left]. After that, it’s gonna (sic) expire.”*

Human resource constraints were equally challenging. One PICT explained that there were only two public health professionals to manage the territory’s entire surveillance portfolio, spanning epidemiologic and entomologic components. In many countries, the same personnel are tasked with managing multiple public health responsibilities, leaving little capacity for disease-specific surveillance activities and limiting ability to maintain routine surveillance or respond to emerging needs.

*“Usually, the hardest [setting] will be the healthcare setting because engaging our health officers to conduct such thing, it’s because they already have multiple things in their work that they do. And to pull them out and teach them on LF and then tell them this is so it’s adding more things to them.”*

Frequent staff turnover, poor institutional memory, and a resultant disconnect among decision-makers was cited as a barrier. To overcome this, participants requested that clear LF PVS guidelines be available to ensure streamlined, clear communication on LF PVS requirements and prevent such knowledge gaps occurring.

*“She already left the service, and I think that’s why there was a delay in conducting our PVS, because there was lack of communication between those people and us at the operational level, and the decision makers.”*

The inherent challenges of geographic isolation, limited transport infrastructure, and high costs associated with the delivery of services in small, remote island states posed significant and complex challenges to establishing effective and timely LF PVS efforts. Participants described the complexity of working with highly dispersed populations over sometimes hundreds of inhabited islands that do not have regular transport links. Limited flights to some PICTs mean that critical equipment and supplies can only be shipped and can take 30 days or more to arrive, with some vessels docking as infrequently as every four to six weeks.

*“The schedule for public ferries and flights are unstable and infrequent—so such challenges and associated costs need to be factored into future PVS plans.”*

Some PICTs identified means to overcome such barriers, e.g., by sending photos of reports via mobile apps. Conversely, in very small PICTs, surveillance is somewhat simplified by the social familiarity and easy mobility of residents. However, even in such settings, timely and targeted responses still depend on adequate financial and human resources and geographic coverage.

The need for ongoing technical support, particularly from external development partners, was a recurring theme. Across all PICTs, reliance on development partners was described as essential for rapid test procurement, operational funding, and/or technical capacity, echoing the dependence on external assistance and the lack of sustainable domestic financing mechanisms. Such dependability on donors and external agencies has become a pervasive issue, with several PICTs expressing concerns that ongoing reliance will undermine long-term sustainability should countries be left to manage surveillance activities without continuous external support. In one PICT, the entire LF programme was financed by external partners, complicating the sustainability of future LF PVS activities should the financial support cease. Similarly, another PICT emphasised that external organisations provided the primary technical guidance and materials, with limited domestic capacity to continue activities without external assistance.

*“There were two nurses that we contracted to collect the blood samples and do the testing. They were contracted, so they were only with us for that length of the project.”*

There was broad consensus on the need to strengthen national and regional capacity for LF PVS through workforce development and technical support, particularly around building a well-trained, cross-disciplinary public health force and ensuring surveillance capacity in remote health facilities. Participants emphasised the value of partnerships with other countries and institutes for technical support, testing, and capacity building (particularly considering their own limited resources). There was strong interest in cross-country collaboration and learning, with calls to share examples of LF PVS plans and research outputs from other PICTs across the region to inform the development of national response plans. Local and regional leadership was recognised as critical to ensuring ownership and sustainability. Participants stressed the importance of using existing community engagement mechanisms and data-driven advocacy to elevate PVS as a national priority. The need for robust evidence and data was also highlighted as critical for convincing political leaders to allocate the necessary resources, an approach that was seen to be effective during COVID-19 and could be replicated for NTDs.

*“The way to do this is to push our political, our leaders, and in order to push them, we will need that data, to the evidence to provide, to provide with them, and that’s all. That’s how we can move. We did that for COVID and we can do that for LF and other NTD.”*

This perspective highlights the perceived importance of evidence-based advocacy in elevating LF and other NTDs on national political agendas, drawing on lessons from the COVID-19 response.

Many PICTs highlighted the importance of aligning PVS activities within existing health structures and surveillance systems to ensure sustainability and reduce costs and programmatic burden. One PICT viewed their current reform of the primary healthcare system as an opportune moment to integrate LF PVS and other NTD-related efforts into broader health system strengthening.

The benefits of routine and integrated surveillance rather than one-off and siloed approaches were highlighted as more sustainable and cost-effective. Participants gave examples of ongoing and well-established surveillance systems in their countries/territories that could be considered as integration opportunities, including vector surveillance for arboviruses (e.g., dengue, chikungunya, and Zika) and robust partnerships with regional organisations, e.g., PacMOSSI (https://pacmossi.org/ [accessed on 1 July 2025]). Leveraging lab capacity to include in-country molecular xenomonitoring was discussed, thereby avoiding the need to outsource to external partners. The integration of LF testing into regular outreach trips by medical ships and mandatory tuberculosis screening were also presented as potential models. Embedding LF in notifiable disease lists, mobilising public health teams for cross-programme surveys, and integrating LF awareness into school health curricula to increase early awareness were also proposed. One PICT stressed the importance of building post-elimination capacity by “piggy-backing” PVS into existing public health data collection and integrating NTD trainings and surveillance (e.g., trachoma and scabies) into a single package to improve efficiency. This, alongside bundling funding opportunities, was considered a means to establish a more cohesive NTD surveillance platform.

*“We can have some funding for training, then we can have a package of training so that, when we train all NTDs, it’s not just for [one disease]. So we could bundle the training and set up an integrated NTD surveillance system.”*

## 4. Discussion

We have consolidated the voices of LF programme managers and representatives from eight LF-eliminated PICTs to identify and understand the implications of the barriers faced in the implementation of PVS and suggested the next steps to overcome these barriers. The experience from these PICTs will provide significant insights to other countries (both regionally and more broadly) in the operationalisation of PVS strategies, ultimately leading to sustained surveillance and maintenance of elimination goals. The framework can be used as a practical planning tool, enabling implementers to distinguish barriers that may be addressed through policy clarification and guidance from those requiring longer-term system investment, financing, and integration. 

We note that as the *WHO 2nd Edition of the LF Monitoring and Evaluation Manual* [7], which includes guidance on the implementation, timing, and duration of PVS, as well as the use of surveillance data, was published after data collection. Thus, the perspectives and recommendations presented here reflect country experiences and challenges prior to the availability of the updated WHO guidance. While the release of the *WHO 2nd Edition of the LF Monitoring and Evaluation Manual* now provides formal technical guidance for LF PVS, the extent to which guidance alone can address persistent implementation challenges remains unclear. In small island health systems, surveillance uptake is shaped not only by technical recommendations, but also by structural, financial, workforce, and political constraints. Understanding why LF PVS was variably adopted prior to the availability of formal guidance and which barriers are unlikely to be resolved by guidance alone is critical to informing feasible, context-specific surveillance strategies.

WHO validation of elimination requires a formal commitment to PVS; our findings indicate that this commitment does not always translate into sustained operational awareness at country level. Participants described how PVS obligations were often poorly communicated beyond the validation process itself, particularly in the context of staff turnover and delayed release of formal guidance. This disconnect highlights the difference between policy-level commitments and their operationalisation within small and resource-constrained health systems.

Although barriers varied in prominence by context, the comparative synthesis highlights a common pattern of structural and system-level constraints across PICTs, reinforcing the need for context-specific, country-led approaches rather than uniform implementation models. A common sentiment from participants was that the absence of clear, timely communication, compounded by frequent staff turnover and resultant poor institutional memory, stalled national action to implement LF PVS. Specific, understandable, and contextually feasible LF PVS guidelines were urgently needed to ensure that countries that had eliminated LF as a public health problem were aware of the requirements to continue surveillance and ensure continuity of activities. A primary barrier to implementing PVS was the uncertainty around the definition of ‘elimination as a public health problem’. As many PICT languages use a single term for both ‘elimination’ and ‘eradication,’ this may have contributed to the presumption that LF surveillance activities were no longer required once transmission had ceased, an observation drawn from this and several other meetings with PICT representatives.

The *WHO 2nd Edition of the LF Monitoring and Evaluation Manual* will go a long way in addressing the ambiguity around PVS implementation, including recommendations on surveillance frequency, reporting mechanisms, and the interpretation of PVS findings to guide next steps. However, the manual still requires some contextualisation at regional and national levels. Examples of successful, regional LF PVS strategies that detail the lessons learned and recommendations should accompany the manual; these can serve as a foundation or template for countries to draw on to develop their own national LF PVS plans. Such an approach enables peer-to-peer learning, knowledge sharing, and national and regional scaling of activities, thereby reinforcing country ownership and developing regional networks and camaraderie to improve the sustainability of LF PVS.

While there are significant barriers to implementing effective LF PVS in PICTs, there are also opportunities to build on existing collaborations and integrate LF surveillance into broader health initiatives. However, it is important to bear in mind that disease priorities are often reactive and subject to resource limitations, pushing LF surveillance down the list of priorities. NCDs continue to dominate the health agenda, particularly in PICTs. Examples of how to sustain LF PVS by integration into well-funded and well-resourced surveillance programmes and surveys should be highlighted in the guidelines. For example, LF PVS was recently and successfully incorporated into a national WHO STEPwise approach to NCD risk factor surveillance (STEPS) survey in Niue [18]. As STEPs surveys are conducted every five years, this could be an ideal platform to integrate PVS for LF (and other eliminated or near-eliminated diseases) at a national level. Advocating for WHO to integrate diseases in the platform would enable population-level disease monitoring, negating the need to identify and allocate separate funding for siloed LF surveillance activities. This will entail decompartmentalising and integrating NCD, infectious disease, and NTD programmes, thereby bringing together surveillance, reporting, training needs, and operationalisation. For example, several WHO regions use integrated primary care models that combine NCD screening with infectious disease case-finding, highlighting the efficiencies of shared surveillance systems.

Crucially, some PICTs are approaching ten years since validation of elimination; thus, conducting PVS is a priority to ensure that the gains made in controlling and eliminating LF are not lost. If LF resurgence occurs in eliminated PICTs, costly re-intervention and surveillance will be required, undoing decades of progress. Since this study was conducted, LF PVS has been conducted in Tonga [16], Wallis and Futuna [19], and Niue [18] and, concerningly, persistent LF transmission was identified in both Tonga and Wallis and Futuna. This emphasises the importance of LF PVS in these settings.

PICTs may struggle to justify continued spending on a disease perceived as “eliminated”. Similarly, obtaining resources from decision-makers for LF PVS is further complicated by the long latency between LF infection and the appearance of clinical signs, which undermines the perceived urgency of what will later become a significant public health concern. Therefore, encouraging national and regional advocacy campaigns targeting policymakers, healthcare providers, and communities should be emphasised about the importance of sustained LF surveillance, particularly around the long-term benefits of PVS in preventing costly resurgence.

An additional challenge highlighted in this study by participants was uncertainty about how to respond to signals of LF transmission. Although the new WHO manual provides some insight into how to interpret positive antigen, antibody, microfilariae, and molecular xenomonitoring results, they offer limited recommendations on appropriate “next steps”. Consequently, LF PVS will require financial investment in laboratory testing, surveillance teams, and data management, as well as strengthened support for data interpretation, logistics, and technical guidance. Identifying sustainable LF PVS strategies, such as integration into existing infectious disease surveillance platforms, will be crucial.

Advice on how to secure and leverage government funding and political commitment for PVS to reduce dependency on external donors would help support the sustainability of surveillance and country leadership. Identifying regional funding mechanisms and supporting country-led funding applications can further increase country ownership and sustainability of LF PVS. Similarly, reducing reliance on external technical assistance through national and regional capacity building is fundamental to ensuring the longevity of LF PVS. Of note, facilitating country ownership is one of the three foundational pillars of *WHO: Neglected Tropical Diseases Roadmap 2030* [9]. If countries remain dependent on external technical and financial support, their ability to sustain LF PVS (as well as surveillance of other NTDs, infectious diseases, and NCDs) independently is weakened, emphasising the need to integrate health programmes. Without country ownership, decision-making and prioritisation may be influenced by external stakeholders rather than local needs, and a lack of domestic accountability may reduce long-term commitment to surveillance efforts.

The establishment of multi-sectoral coordination between government agencies, research institutions, and community organisations, and developing governance structures where national and regional health authorities take ownership of PVS programmes, can reduce reliance on external technical assistance and has the potential to lead to national and regional leadership and PVS programme governance. National policy changes could be strengthened by aligning them with regional frameworks that already guide public health action in the Pacific, such as the Pacific Public Health Surveillance Network, the Healthy Islands Vision, and (globally) the WHO NTD Roadmap 2030. Such alignment would provide established structures for integrated surveillance, cross-programme coordination, and resource mobilisation. Additionally, encouraging collaboration and knowledge exchange within the region can create a support network for national and regional surveillance frameworks that can be adapted to PICT contexts and harmonise surveillance strategies.

While translating identified barriers into implementation strategies is a critical next step, accomplishing that in a prescriptive manner was beyond the scope of this study. The barriers identified operate at multiple levels and are deeply shaped by country-specific governance structures, workforce capacity, financing mechanisms, and sociocultural contexts. Developing practical strategies will therefore require in-depth, country-led analyses and engagement with national stakeholders to ensure feasibility, acceptability, and sustainability. Rather than proposing uniform solutions, the value of this study lies in providing a shared conceptual framing that can be used by WHO, regional partners, and countries as a diagnostic tool to identify priority constraints and guide subsequent, context-specific planning processes for LF PVS.

## 5. Conclusions

In PICTs, elimination was frequently viewed as an endpoint rather than a transition to sustained vigilance. Although WHO guidance on PVS has since been released, our findings reflect country experiences at a time when such guidance was not yet available. These findings point to the importance of pragmatic, timely, and contextually relevant LF PVS guidelines alongside proactive capacity building, stronger communication strategies, and integration of LF surveillance into national priorities, plans, and budgetary commitments. Identifying dedicated national and international funding mechanisms and regional support to alleviate the financial strain on PICTs tasked with LF PVS is needed to ensure the sustainability of LF PVS. Our study further emphasises highly contextual operational challenges for disease surveillance in PICTs. For sustainable and equitable surveillance, strategies must be tailored to the realities of both high-cost, hard-to-reach islands and smaller, more dispersed populations. This further reinforces the importance of integration and resource optimisation for LF PVS to succeed.

## Figures and Tables

**Table 1:** Feedback from online screening questionnaire detailing current post-validation surveillance activities for lymphatic filariasis in the eight Pacific Island Countries and Territories that have eliminated lymphatic filariasis as a public health problem. Year of WHO validation is included to provide contextual information on time since elimination.

Country/Territory (Year WHO Validation Received)	When Were Your Most Recent LF Surveillance Activities Conducted? * Please Choose from (i) Within the Last Year; (ii) Less Than 3 Years Ago; (iii) More Than 3 Years Ago; and (iv) Unsure/Don’t Know	Please Describe What LF Surveillance Activities Were Conducted?	This Year (2024), Do You Plan to Conduct Any LF Surveillance Activities?
Niue (2016)	More than 3 years ago	School-based TAS	None/unsure
Tonga (2017)	More than 3 years ago	School-based TAS	Yes—planned operational research study with The University of Queensland
Vanuatu (2016)	More than 3 years ago	TAS in 2012	Since 2021, Vanuatu NTD program in collaboration with Kirby Institute (Australia) and WHO have been conducting a serosurveillance study to test for Ab against NTDs (including LF) in 3 yaws priority provinces. However, 3 provinces (where previously highest LF prevalence) are not included due to lack of funding.
Kiribati (2019)	More than 3 years ago	None/Unsure	Community-based vector control programs and clean-up campaign.
Republic of the Marshall Islands (2017)	Unsure/Don’t know	None/unsure	None/unsure
Wallis and Futuna (2016)	Within the last year	Eosinophilia surveillance since October 2023 following the diagnosis of 2 LF cases in Futuna.	Further case investigation in schools and colleges; voluntary screening of population aged 6 years and over; molecular xenomonitoring around suspected hotspots.
Cook Islands (2016)	More than 3 years ago	School-based TAS	Targeted surveillance in suspected LF hotspots.
Palau (2017)	More than 3 years ago	TAS and migrant worker screening.	None/unsure

* Survey distributed in 2024. Ab = antibody; LF = lymphatic filariasis; NTD = neglected tropical disease; TAS = Transmission Assessment Survey; WHO = World Health Organization.

## Data Availability

The data that support the findings of this study are available on request from the corresponding author, H.L.S.L. The data are not publicly available as they contain information that could compromise the anonymity of research participants.

## Abbreviations

The following abbreviations are used in this manuscript:

Ab	Antibody
Ag	Antigen
COR-NTD	Coalition for Operational Research on Neglected Tropical Diseases
GPELF	Global Programme to Eliminate Lymphatic Filariasis
HREC	Human Research Ethics Committee
LF	Lymphatic filariasis
MDA	Mass drug administration
MMDP	Morbidity management and disability prevention
MOH	Ministry of Health
NCD	Non-communicable disease
NTD	Neglected tropical disease
NVivo	Qualitative data analysis software (QSR International)
PacELF	Pacific Programme to Eliminate Lymphatic Filariasis
PacMOSSI	Pacific Mosquito Surveillance Strengthening for Impact
PICTs	Pacific Island Countries and Territories
PVS	Post-validation surveillance
STEPs	STEPwise approach to NCD risk factor surveillance
TB	Tuberculosis
UQ	The University of Queensland
WHO	World Health Organization

## Appendix A

**Interview Guide**

**Research Title:** Post-Validation Surveillance of Lymphatic Filariasis in the Pacific Island Countries and Territories
**Researcher(s):**

- Dr Harriet Lawford, Research Fellow, The University of Queensland
- Dr Adam Craig, Senior Research Fellow, The University of Queensland
- Professor Colleen Lau, NHMRC Fellow and Professorial Research Fellow, The University of Queensland

**Verbal informed consent.**
**Notification that participant can withdraw consent at any time, including after the interview.**
**Introduction**
Thank you for your interest in participating in this research project. We are talking to health representatives from Pacific Island Countries and Territories who have achieved elimination of lymphatic filariasis (LF) to understand what post-validation surveillance (PVS) activities are being conducted in your country/territory.
Please answer a few introductory questions about your work on LF:

- What is your role in the [health sector/MOH]?
- How long have you been in this role?
- Can you describe your work in LF surveillance to date?
- When was [your country] certified as having eliminated LF?
- Are you aware of any PVS activities that are being conducted in [your country/territory]?
  o When and where were these activities conducted?

**For countries with PVS activities**

- Is LF resurgence a concern in [your country/territory]?
- Can you describe the PVS activities that have been conducted since [your country/territory] was certified as having eliminated LF?
  Prompts:
  o Were activities targeted to a specific demographic group?
  o Were activities targeted to a specific area?
  o What tools have been used to test for LF?
- Official WHO guidelines for PVS are currently under development but are likely to include two or more of the following: molecular xenomonitoring of mosquitoes; integrated serological surveillance; health facility-based surveillance; targeted surveys. If any of these activities being conducted in your country, can you describe the barriers/facilitators around this/these surveillance methods?
  Prompts:
  o If not, have you considered these surveillance methods?
  o What resources would be required for each of these activities?
  o What are the barriers to conducting these activities in your country? e.g., technical expertise, funding, human resources, logistics…
  o Has your country conducted PVS using other strategies (i.e., not the four mentioned above)?
  o Community acceptance?
- Who has coordinated PVS activities in [your country/territory]?
  Prompts:
  o Were external bodies consulted?
  o Do you receive support outside of [your country/territory] to conduct PVS activities?
- If PVS has been conducted, has any LF transmission been found?
- What is the process for reporting, managing, and following up LF cases if they are identified?
- What resources are required to conduct PVS activities?
  Prompts:
  o Additional funding required?
  o Supply of diagnostics and treatment?
- What are the barriers to conducting PVS in [your country/territory]?
- What are the facilitators to conducting PVS in [your country/territory]?

- **For countries without PVS activities**

- Is LF resurgence a concern in [your country/territory]?
  o Are there any particular regions/districts of concern?
  o Are there any subpopulations (age groups, demographics [migrants, occupational groups, international travellers]) of concern?
- Is PVS an activity you believe is important to conduct in [your country/territory]?
- What resources and/or support would be required to conduct PVS activities in [your country/territory]?
  Prompts:
  o Additional funding required?
  o Technical expertise
  o Supply of diagnostics and treatment?
  o Logistic challenges?
  o Guidelines to develop strategy/protocol?
  o Programmatic support?
- What would you consider a challenge/barrier to conducting PVS in [your country/territory]?
  o Community acceptance?
- Do you believe PVS activities are feasible in [your country/territory]?
- What resources do you feel would be required to conduct PVS activities?
  Prompts:
  o Additional funding required?
  o Supply of diagnostics and treatment?
- What are the barriers to conducting PVS in [your country/territory]?
- What are the facilitators to conducting PVS in [your country/territory]?
